# The use of leaded paints in an urban neighborhood in Quito, Ecuador: A case study

**DOI:** 10.1038/s41598-026-48544-w

**Published:** 2026-04-25

**Authors:** Hugo Navarrete, Gabriela S. Yánez-Jácome, Pamela Y. Vélez-Terreros

**Affiliations:** 1 Centro de Estudios Aplicados en Química (CESAQ), Pontificia Universidad Católica del Ecuador, Nayón, 170530 Ecuador; 2 Facultad de Ciencias Exactas, Naturales y Ambientales, Pontificia Universidad Católica del Ecuador, Quito, 170525 Ecuador

**Keywords:** Flame atomic absorption spectrophotometer, Field painted spots, Lead, Threshold limits, Method validation, Portable X-ray fluorescence analyzer, Environmental sciences, Environmental social sciences

## Abstract

Lead has been used in paints since ancient times, posing significant public health and environmental risks. In 2012, a maximum permissible concentration of 600 ppm of Pb in paints was adopted in Ecuador, however since 2024 a new maximum limit of 90 ppm was stablished, with an implementation period of 30 months. In this regard, the present study aimed to present for the first time a preliminary screening related to the presence of Pb in paints used in exterior walls, public spaces, and streets in a densely populated area in Quito-Ecuador, analyzing 171 painted spots in situ through a portable X- Ray Fluorescence (pXRF) analyzer. Pb content was compared to the former and latest maximum threshold limits. Results varied from < LOD (5 ppm) to 45,216 ppm in painted spots with different colors and paint uses, in which 40.4% of samples exceeded the 600 ppm limit. Regarding colors and paint use, yellow, orange, green, and two samples of red, and road marking and metallic paint presented the highest Pb values. This preliminary study shows that regulation for the use of lead-based pigments in paints in Ecuador continues to face significant regulatory and enforcement challenges, exposing populations to harmful Pb levels.

**Supplementary Information:**

The online version contains supplementary material available at 10.1038/s41598-026-48544-w.

## Introduction

Lead (Pb) is a naturally occurring metallic element that has played a significant role since antiquity, as one of the most used metals in numerous civilizations^1,2^. Historically, Pb was used in the manufacture of pipes for urban water distribution systems, drainage conduits, dishware, coins, and cosmetics. It was also used to produce lead acetate, a highly toxic compound regarded as the first artificial sweetener, intended to enhance the flavor of wine and various foods^3^. Besides the use of the metallic form, the pigments based on Pb have a profound impact along the human history and across the continents^4–9^.

Despite their well-documented toxicity, lead compounds have occupied a prominent place in the history of pigments, valued for their vivid hues and exceptional durability. Among the earliest known Pb-based pigments is “lead white” (lead carbonate, PbCO₃) which was extensively used in ancient Greece, Rome, and later in European art. Its brilliant whiteness made it especially suitable for frescoes, manuscripts, and oil paintings^10^. Another prominent lead-based pigment is “red lead” (minium, Pb_3_O_4_) used in illuminated manuscripts and early Chinese ceramics for its vibrant orange-red hue^11^.

Although strict regulations govern the use of Pb-based pigments, they continue to be widely employed and represent a substantial risk to both public health and the environment. These risks are particularly concerning due to lead’s well-documented neurotoxicity and its long-term detrimental effects on human health^12^. Mahmoodi et al. (2021)^13^ pointed out that chrome yellow (PbCrO_4_) inside yellow pavement marking paints (with an annual global production of approximately 90,000 tons) is the major source of Pb present in atmospheric dust, soil, groundwater, and surface water resources. Despite the demonstrated negative health effects and regulatory measures aimed at restricting the use of Pb-based pigments, they are still widely used. Recent research has explored the development of alternative formulations designed to replicate or enhance their remarkable stability^14^.

From a regulatory standpoint, most countries worldwide have enacted legislation to control the presence of Pb-pigments in various products, including food, paints, and toys^15–17^. In high-income countries, such as the United States and the member states of the European Union (EU), the use of lead-based pigments in household paints, including architectural and decorative coatings, has been banned. However, industrial paints are frequently exempt from these restrictions, and lead-based formulations continue to appear on the market shelves of both developed and developing countries^18^. A review of Pb concentrations in paints revealed dangerously high levels in products from South Africa, Thailand, and Brazil, underscoring the urgent need for stricter regulatory controls^17^. Paradoxically, Pb-based pigments continue to be widely manufactured in certain high-income countries and are subsequently marketed and used across many developing countries^19^.

While high-income countries have made significant progress in reducing Pb in paints and other products, Ecuador and many Latin American nations continue to struggle with regulation enforcement. Across Latin America and the Caribbean, countries such as Ecuador have established regulatory frameworks that limit Pb concentrations in paints to 600 ppm, while others, like Paraguay, have adopted stricter thresholds of 90 ppm^20^. However, Pb concentrations in decorative paints and road marking paints often exceed these limits, with some samples showing alarming values as high as 236,000 ppm^21^. In 2024 Ecuadorian Government decided to reduce the limit of Pb to 90 ppm in all types of paints^16^.

In light of this context, and the increasing concern regarding the ongoing use of lead Pb-based pigments in Ecuadorian paints, this study aims to conduct a preliminary screening of Pb concentrations in paint applied to exterior walls, public areas, and road markings within a densely populated sector of Quito, Ecuador. Pb levels were subsequently evaluated against both the former and the most recent maximum permissible limits for Pb in paints established by the Ecuadorian Standardization Service (INEN, by its acronym in Spanish)^22^. To the best of our knowledge, this is the first study conducted in Ecuador addressing this issue. It also evaluates the extent to which current regulatory frameworks governing paints in Ecuador are being effectively implemented.

## Materials and methods

### Study area

Considering the severe health impacts associated with lead (Pb) exposure, the present research is focused in a study area of approximately 2 km², designated as the “Universities Sector” in Quito. This area encompasses four major Universities, two elementary schools, nurseries, multiple hotels, and several public administrative offices. Approximately 60,000 people move through this area daily, excluding those traveling through the zone in buses or private vehicles, and permanent residents. We also selected this area due to a major urban program that converted several streets into pedestrian-friendly corridors and promoted public use of space for artistic interventions, including themed murals and street art; consequently, substantial quantities of paint have been applied over the last five years.

**Fig. 1:**
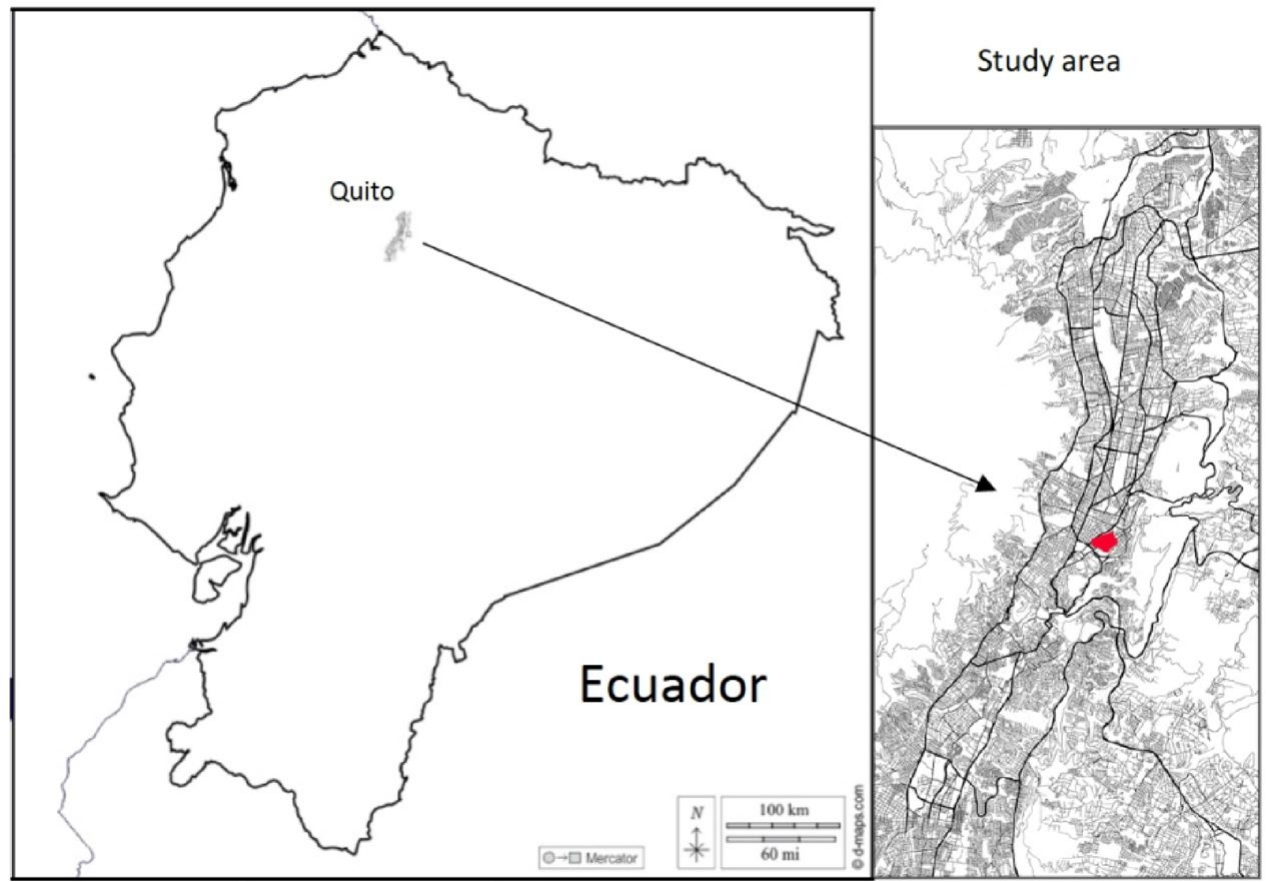
Sampling area highlighted in red, covering ca. 2 km^2^. This zone is known as the Universities sector. Quito - Ecuador. Map of Ecuador from https://www.d-maps.com/carte.php? num_car=3385&lang=en; map of Quito based on vectorstock_47823343, purchased on February 2026.

The selected study area represents a small fraction of Quito; however, it has a significative pedestrian and vehicular traffic, considering the distribution of various institutions without order and structure, and the lack of sufficiently organized transport systems in the area, as well across the whole city^23,24^(Fig. 1). No official authorization for this study was required since all the sampling sites were located in public areas and no commercial brands or institutions names were involved.

## Lead content and analyses

The Pb concentration in paints applied on external walls, roads, and sidewalks was analyzed using a portable Olympus Vanta Series C (Evident Scientific) X-Ray Fluorescence (pXRF) spectrometer, with LP-VCA OLYMPUS method for Pb in paints. The pXRF is a rapid, non-destructive, and semi-quantitative analytical technique suitable for detecting Pb and other metals in paint samples without altering their structure^25^. Seney et al. (2025)^26^ demonstrated that this technique is a reliable, rapid, and cost-effective method for screening the presence of Pb in dry paint samples.

In the study area, 171 painted sites were randomly selected and analyzed in situ. Measurements were performed directly on painted surfaces after cleaning with high-quality reagent water obtained from a Genie 5 Direct-Pure Water System (Rephile, Shanghai, China), using a laboratory wash bottle and paper towel. This procedure was performed to remove dust, soil, debris, and traffic-related dust. Surface was let dry and this procedure was performed in every painted spot using a new paper towel.

For Pb determination in selected painted spots, the analyzer was kept straight to the surface to minimize geometric effects. The instrument was set to operate with a three-beam sequence: (a) 40 kV for 15 s, (b) 10 kV for 15 s, and (c) 50 kV for 10 s, without collimation. The calibration of pXRF for Pb and As in situ analysis was performed using calibration algorithms loaded into the instrument software as factory set internal calibrations^27,28^. Additionally, a certified reference material CRM013-50G (Trace Metals–Paint Chips, Sigma-Aldrich RTC, WY, USA) was analyzed through pXRF to verify the accuracy of the technique (Table 1).

To support reliability of pXFR results compared to laboratory-based methods, and corroborate in situ Pb findings, Pb content was quantified through Flame Atomic Absorption Spectrophotometry (FAAS). A total of three yellow paint samples were acquired from local stores during the final quartely of 2024. Paint containers were transferred to the Centro de Estudios Aplicados en Química (CESAQ) for sample preparation and analysis through FAAS. Samples for the present study were commercially available to public; thus, permits were not necessary for their analysis. Brands and manufacturers were handled under confidentiality.

Each paint sample was homogenized and applied with a brush onto individual surfaces of approximately 230 cm², which were allowed to dry prior to pXRF analysis. For FAAS analysis, a portion of each paint was poured into Petri dishes and left to dry to obtain paint chips. Subsequently, 0.5 g of dried paint chips were weighed into MARSXpress Teflon vials, to which 10 mL of concentrated nitric acid (HNO₃) were added for digestion (trace metal grade, Certified ACS, Fisher Chemical, Canada, CAS# 7697–37–2). Acid digestion was carried out using a MARS 6 microwave (CEM Corporation, Matthews, NC, USA) under the following conditions: 1030–1800 W, 180 ◦C, and 15-min holding time. The digested solutions were filtered and diluted to a final volume of 50 mL with high-purity reagent water (resistivity of 18.2 MΩ·cm at 25 °C). Pb concentrations were determined using a Flame Atomic Absorption Spectrophotometer (PinAAcle 900T, PerkinElmer Inc., Waltham, MA, USA).

Calibration for FAAS was performed using an external multipoint curve obtained from serial dilutions of a NIST-traceable certified Pb stock solution (1,000 ± 10 mg L⁻¹; Inorganic Ventures, USA). The calibration curve exhibited a coefficient of determination (*R²* > 0.99). The instrumental limits of detection (LOD) and quantification (LOQ) were calculated from the standard deviation of blank signals (*n* = 10), yielding values of 0.5 ppm and 1.7 ppm, respectively.

### Statistical analysis

Lead content in paints on site was quantified using pXRF, and the resulting data were exported from the Olympus Vanta software. Descriptive statistics, including median, minimum, and maximum Pb concentrations, were calculated using Microsoft Excel 2016, the Real Statistics Data Analysis ToolPak (Microsoft Corporation, Albuquerque, NM, USA), and R software^29^. Concentrations of Pb and other analytes were expressed in parts per million (ppm), equivalent to milligrams per kilogram (mg·kg⁻¹).

The limit of detection (LOD) for Pb of VANTA C series device is 5 ppm^30^. LOD refers to the minimum concentration of an element detectable with reasonable confidence. As Pb levels for some samples were below the LOD, and others were extremely high, we report the median values to avoid skewed data.

The preliminary analysis of the database showed that none of the quantitative variables achieved statistical assumptions to perform parametric statistical analysis, as some metal concentrations were below the LOD across analyzed painted spots. To measure the correlation between Pb and As concentrations in all analyzed paints, we determined Pearson’s correlation coefficient (r).

Also, a principal component analysis (PCA) was performed with z-score standardized and centered data to visualize differences in metal concentrations among the painted surfaces and paint´s color. PCA was performed only for samples (*n* = 86) where metal concentrations were ≥ 5 ppm.

## Quality assurance

To evaluate the analytical performance of both Pb determination techniques, repeatability and intermediate precision were examined. Repeatability (RSDr) was determined from triplicate analyses per sample, while intermediate precision (RSDp) was assessed from triplicate sets performed on three different days by distinct analysts. Accuracy was confirmed through recovery analyses using the certified reference material CRM013-50G (Trace Metals – Paint Chips, Sigma-Aldrich RTC, WY, USA). The mean recovery values (mean ± SD) for Pb measured by pXRF and FAAS are summarized in Table 1.

After validation, a one-way analysis of variance (ANOVA) was conducted to assess the presence of statistically significant differences among the mean values derived from each analytical technique.

For interpretative purposes, the results were compared with the maximum permissible concentration established in the Ecuadorian standard, which sets a threshold of 600 ppm of lead in architectural paints and related surface coatings^22^. Additionally, our findings were contrasted with the updated version of the Ecuadorian regulation, which establishes a new mandatory limit of 90 ppm of Pb for all types of paints. However, this revised standard will only become effective in 2027, thirty months after its publication in October 2024 ^16^.

**Table 1:** Pb content comparison between pXRF and FAAS techniques in three different paints.

FAAS				pXRF			
Pb content (ppm)	RSDr^*^ (%)	RSDp^*^ (%)	CRM Recovery (%)	Pb content (ppm)	RSDr^*^ (%)	RSDp^*^ (%)	CRM Recovery (%)
31,691	0.67	8.49	106.1	28,206	1.17	1.95	82.8
51,375	0.39	7.64		44,874	0.97	1.00	
102,301	0.52	15.85		76,957	1.58	1.66	

*RSDr: repeatability

*RSDp: intermediate precision

## Results

### Lead content in painted spots

A total of 171 painted sites within the study area were analyzed. Regarding Pb presence, 23 sites showed undetectable levels, while the remaining 148 exhibited concentrations ranging from 5 ppm to 45,216 ppm (Supplementary Table S1). Among the samples with detectable Pb (≥ 5 ppm), only 45.3% were below the current regulatory limit of 90 ppm, whereas 54.7% exceeded 600 ppm. The median Pb concentration reached 8,079 ppm, approximately 13 times higher than the former maximum permissible limit (Table 2).

**Table 2:** Pb minimum, median and maximum content in 171 painted spots analyzed in the “Universities sector” in Quito, Ecuador, by XFR.

Number of analyzed painted spots	Lead range (ppm)	Lead content (ppm)			
		Minimum	Median	Maximum	
23	< LOD	< LOD	< LOD	< LOD	
67	≥ 5 - <90	5	11	75	
12	≥ 90 - <600	135	185	566	
69	≥ 600	611	8,079	45,216	
Total: 171					

LOD: limit of detection.

**Fig. 2:**
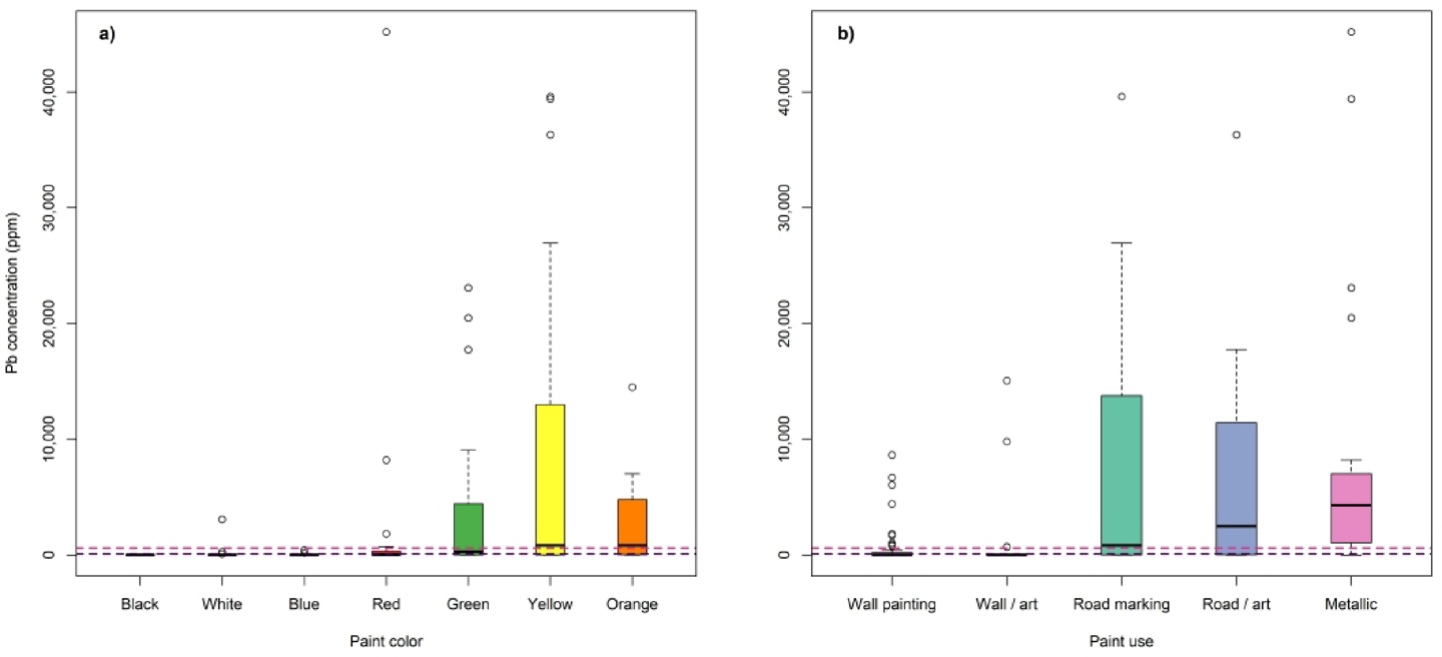
(**a**) Lead content in applied paints according to color. (**b**) Lead content determined in different applied surfaces. Pink and purple doted lines show the former and the latest threshold limits of Pb in paints (600 ppm and 90 ppm, respectively), according to INEN (INEN, 2012; MPCEIP, 2024).

The distribution of Pb content varied significantly by color category (Supplementary Table S2). Orange, yellow, green, and certain red paints exhibited markedly elevated Pb levels, while white, blue, and black paints contained comparatively lower concentrations (Fig. 2a). When classified by use type, metallic, street/art, and road marking paints displayed the greatest Pb presence, followed by wall/art applications, whereas architectural paints generally contained minimal or undetectable Pb (Fig. 2b; Supplementary Table S3). Notably, 54.4% of road marking and 76.2% of metallic paint samples surpassed the 600 ppm limit, with yellow paints representing the most Pb-intensive subset.

Sampling encompassed both new and aged painted surfaces, with Pb concentrations spanning from below the detection limit (< LOD) up to 45,216 ppm (Supplementary Table S4). Based on visual inspection, paints were categorized by apparent age: older coatings (applied over one year prior) showed signs of weathering such as discoloration, opacity, and flaking, while newer paints retained a glossy and homogeneous appearance (Fig. 6 and 7). Remarkably, even freshly applied coatings revealed elevated Pb levels, reaching up to 39,414 ppm in metallic paints and 39,613 ppm in road-marking paints.

**Fig. 3:**
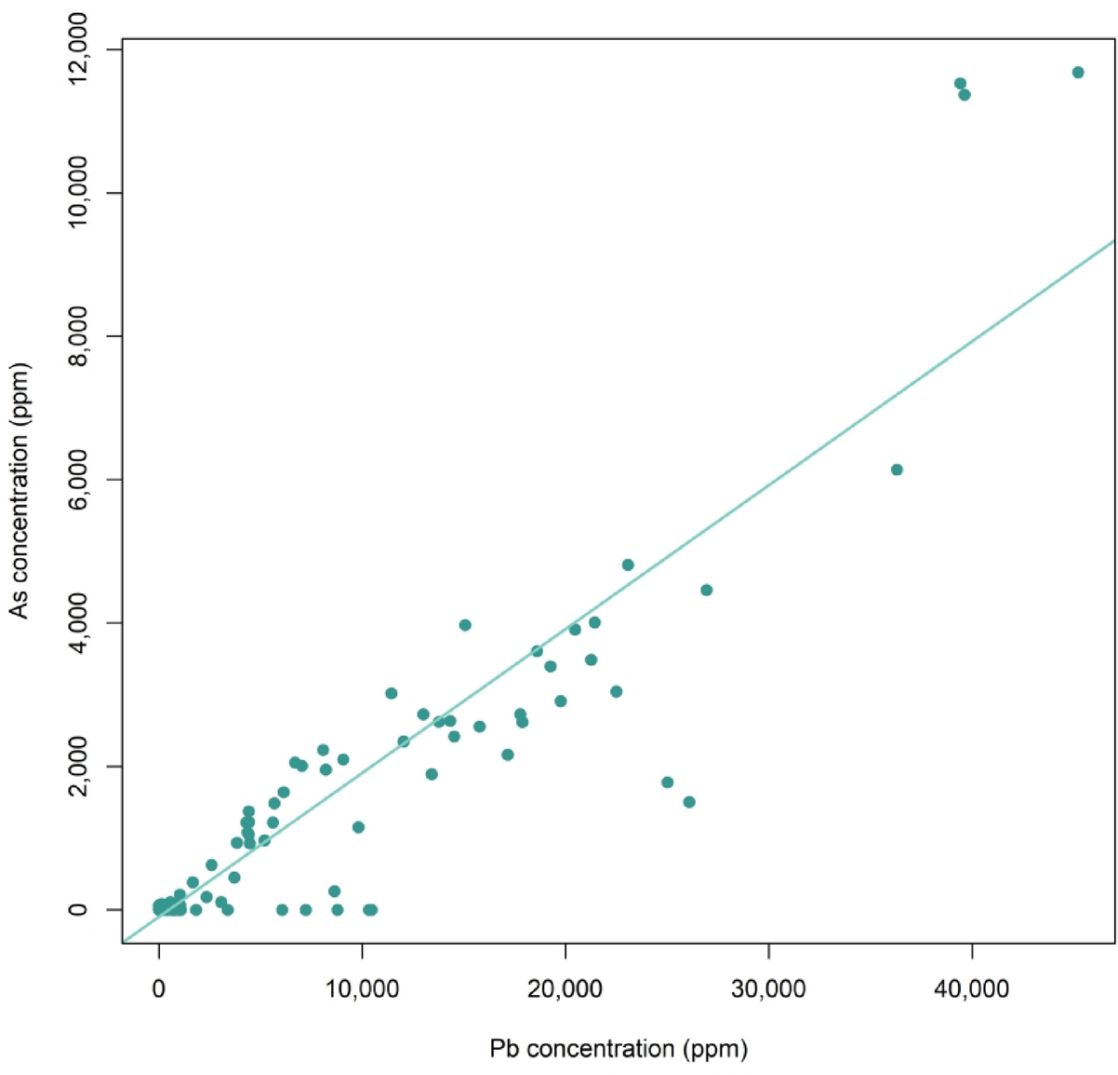
Lead and arsenic correlation in analyzed sample painted spots.

In addition to Pb, unexpectedly, pXRF analysis revealed the presence of arsenic (As) in 55.6% of the analyzed paint samples, with a median concentration of 256 ppm. The trend line showed a significant positive correlation between As and Pb concentrations (*r* = 0.921), as illustrated in Fig. 3. The highest As levels were detected in metallic paints (median = 968 ppm) and road-marking paints (median = 2,255 ppm), and road / art (median = 579 ppm). In contrast, paints samples negative for Pb and used for road markings, wall painting, and wall/art applications generally exhibited non-detectable As levels.

**Fig. 4:**
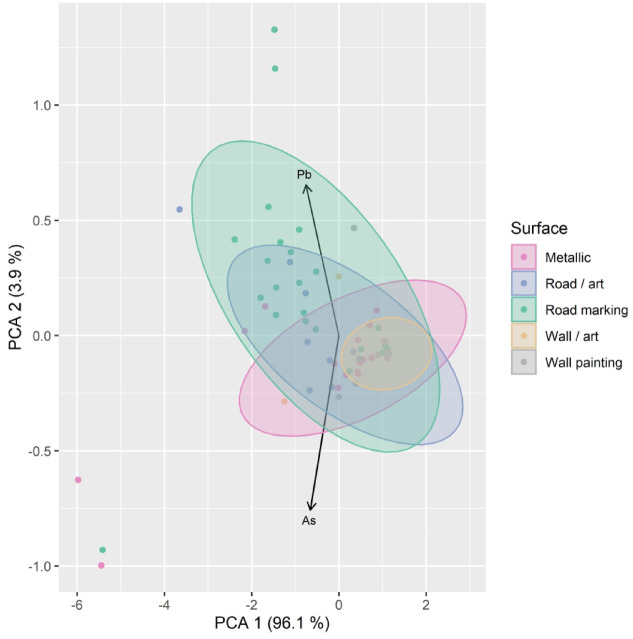
Principal component analysis (PCA) correlation plot showing Pb and As concentrations in the analyzed painted surfaces. Analysis was performed on samples (*n* = 86) with metal levels above the limit of detection (≥ 5ppm).

**Fig. 5:**
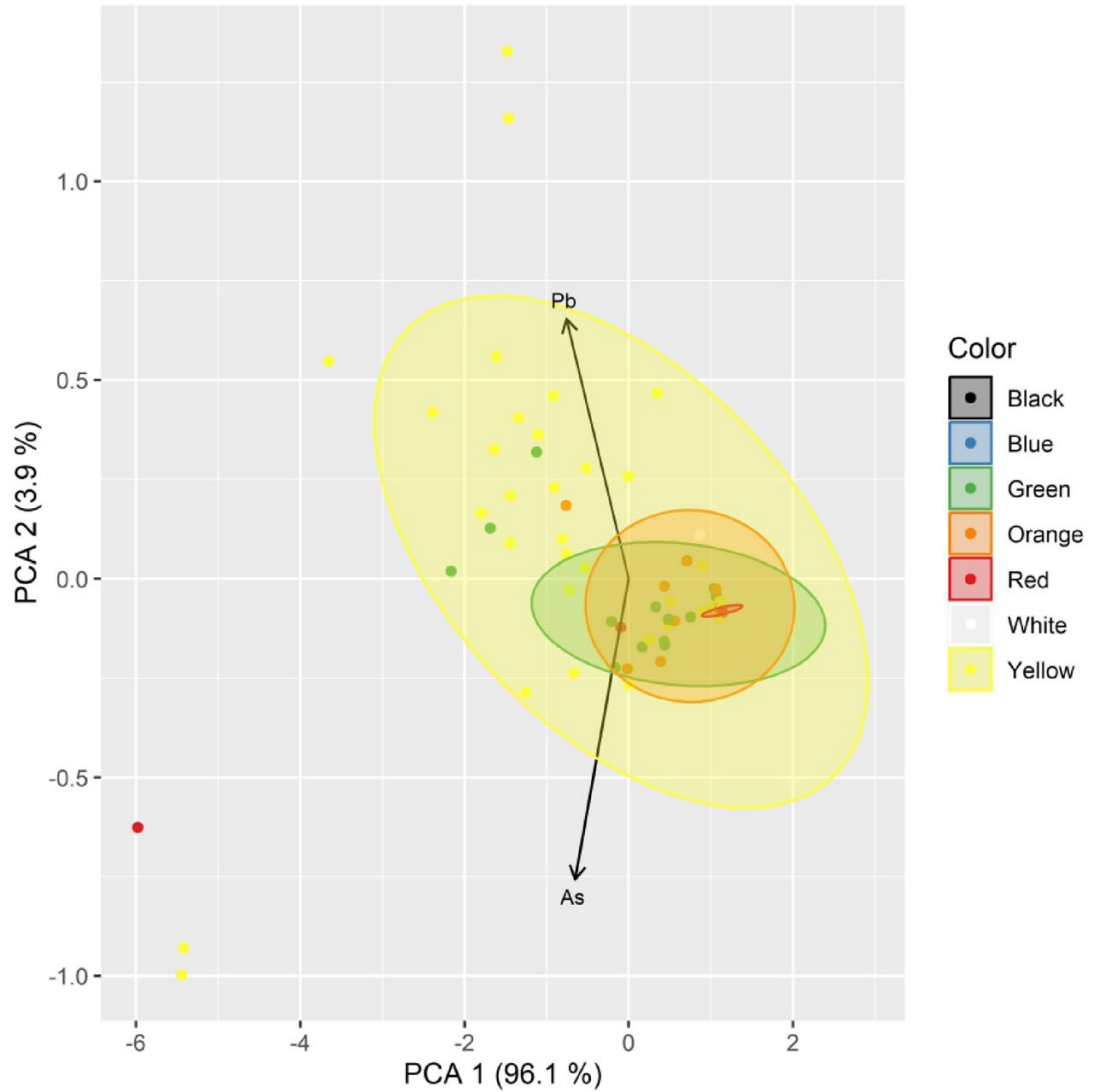
Principal component analysis (PCA) correlation plot showing Pb and As concentrations by paint´s color. Analysis was performed on samples (*n* = 86) with metal levels above the limit of detection (≥ 5ppm).

For the PCA analysis (Figs. 4 and 5), component 1 showed 96.1% of data variation. A clear clustering and overlapping for painted samples were found, highlighting high levels of Pb and As among different surfaces and colors (mainly yellow, orange and green). Five samples of yellow and 1 red paint used in road marking, road/art, and metallic surfaces, were dispersed from the cluster.

**Fig. 6:**
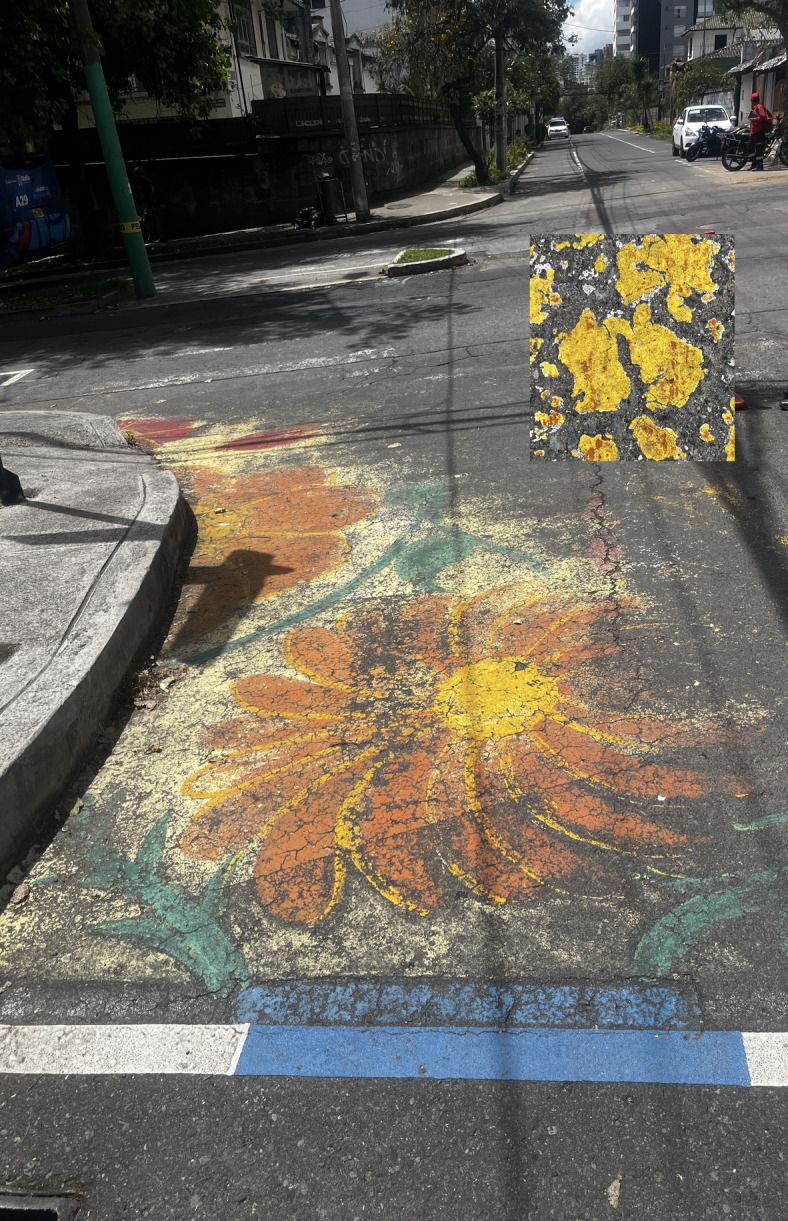
treet painting categorized as “old”. Visible deterioration signs as discoloration, opacity, and flaking. Credit: Hugo Navarrete.

**Fig. 7:**
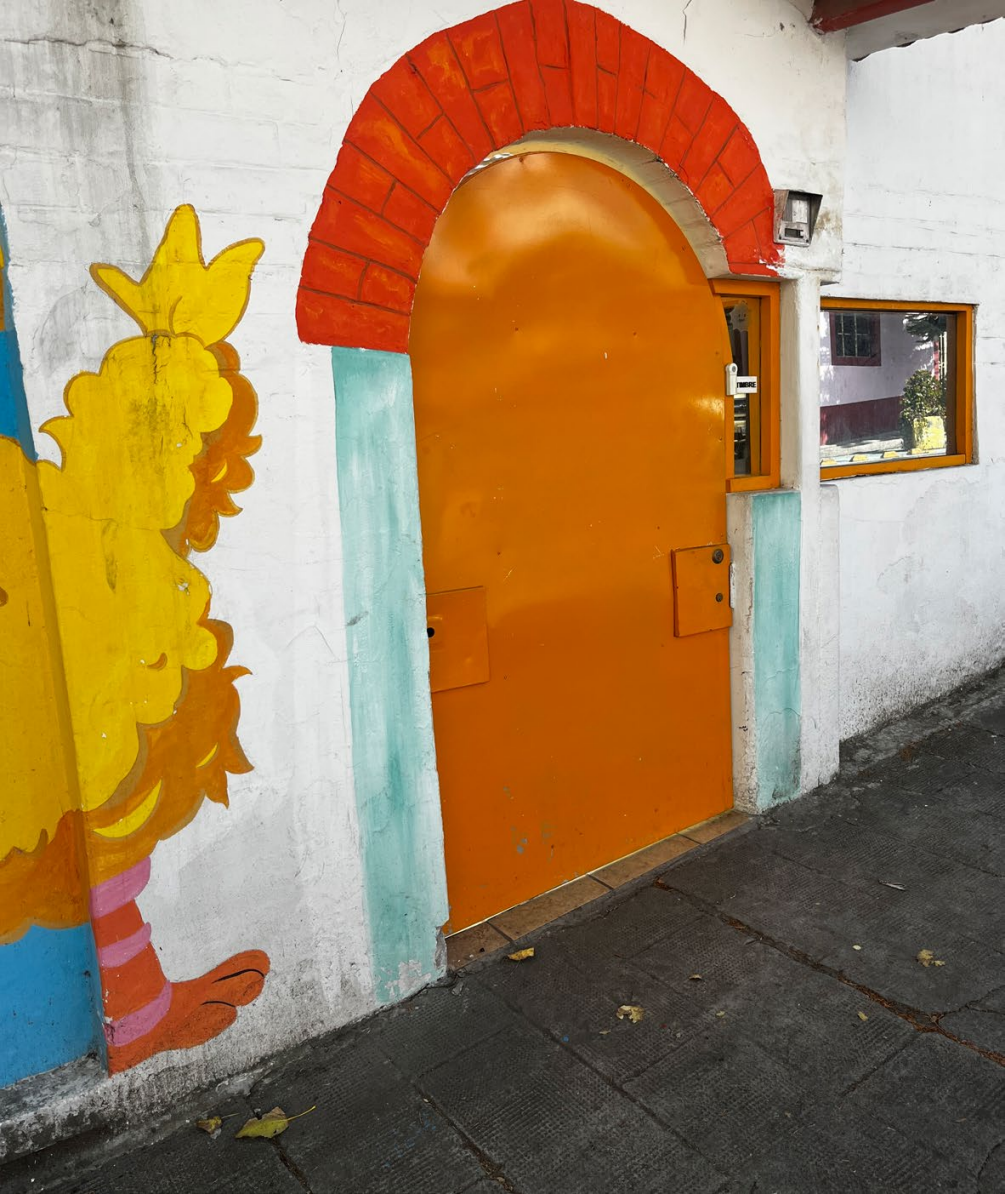
Street painting categorized as “new”. Paint with shiny, smooth and uniform appearance. Credit: Hugo Navarrete.

**Fig. 8:**
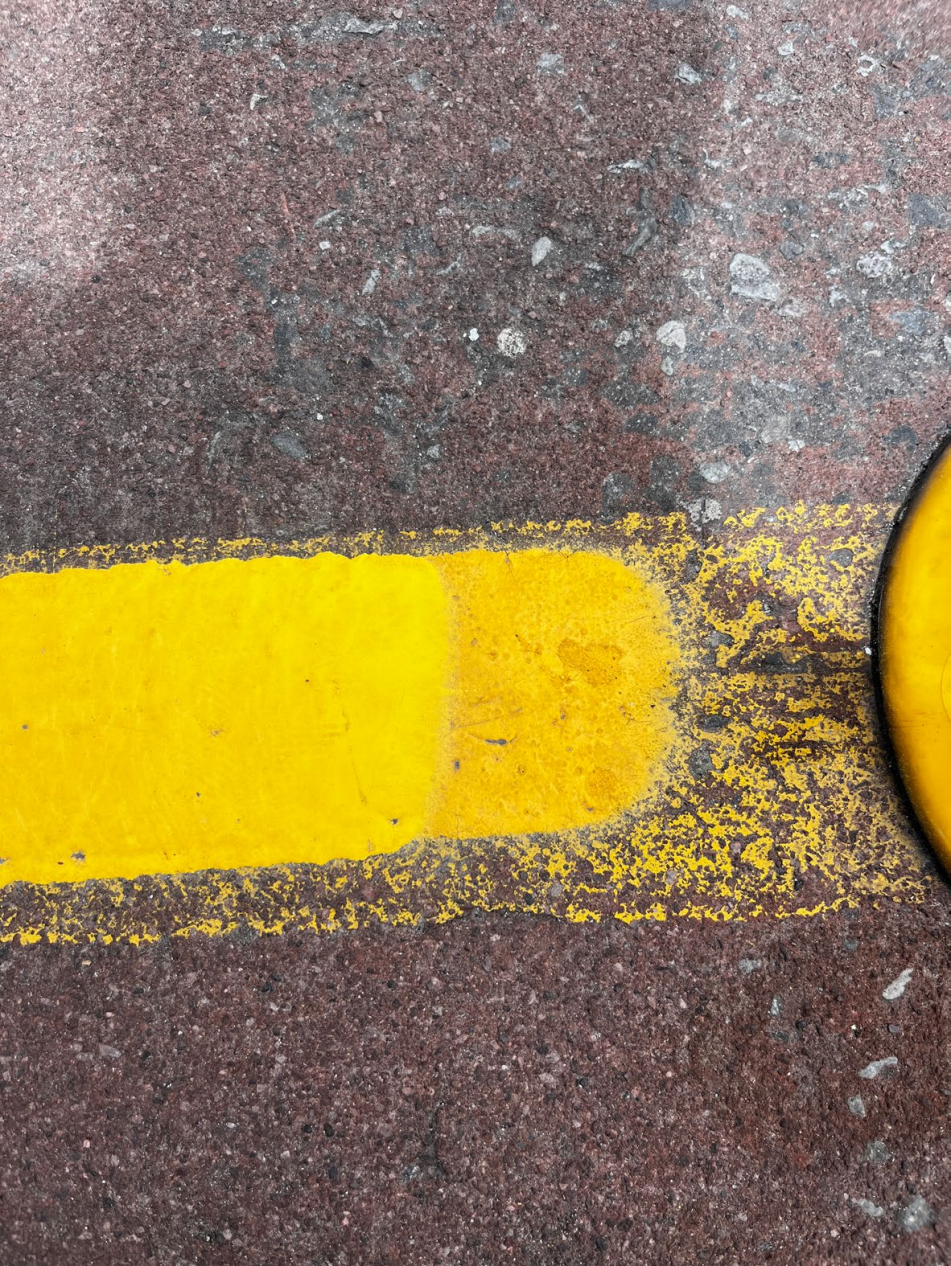
Traffic marking paint with visible repainting layers. Credit: Hugo Navarrete.

## Comparison between pXRF and FAAS measurements

Portable XRF and FAAS measurements were compared by calculating the relative standard deviation (RSD) from three replicate analyses of each purchased paint sample. The results showed a maximum variation of 15.85%, which falls within the acceptable precision threshold for replicate analyses (< 25%) established by the U.S.^31^. Furthermore, recoveries obtained from the certified reference material (CRM013-50G, Trace Metals – Paint Chips) were 106.1 ± 13.5% for FAAS and 82.8 ± 4.1% for pXRF. These recovery values were in good agreement with the certified concentration range of the CRM (369–917 ppm).

No significant differences (ANOVA, *p* < 0.001) were found between the Pb content comparing pXRF and FAAS measurements, therefore for the present study the pXRF was used as reliable, faster and non-destructive method for screening Pb in paint on different surfaces, which is essential for large-scale urban assessments.

Even though limitations of pXFR on potentially heterogeneous objects are known, including nondiscrimination of multilayer samples and relatively high detection limits^32^, for the present study, these issues were controlled. The VANTA C device used only penetrates a few millimeters in the material, being most suitable for surface-level analysis, allowing the determination of Pb content in the painted layer under study. Moreover, the XFR device is equipped with a silicon drift detector letting analyze rough surfaces^30^. On the other hand, the LOD of the portable equipment was significantly lower (5 ppm) than the established Pb threshold limit of the Ecuadorian regulation (90 ppm).

## Discussion

The persistence of lead-based paints in Ecuador can be attributed to multiple interrelated factors: notably weak regulatory enforcement, low levels of public awareness, and the economic appeal of cheaper Pb-based pigments. While the United States and the European Community have implemented strict regulatory frameworks reinforced by systematic compliance monitoring and public outreach, enforcement across much of Latin America remains fragile, despite the existence of relatively comprehensive legislative instruments.

In Ecuador, the regulation limiting Pb content in paints to 600 ppm was established in 2012^22^. However, in October 2024, a new regulation was enacted^16^, reducing the maximum permissible Pb concentration to 90 ppm for all types of paints and establishing an implementation period of 30 months to comply with these stricter limits. Given the persistent use of Pb-based pigments, there appears to be no compelling technical justification for such a delayed implementation. Urgent measures are therefore required to ensure the immediate enforcement of this updated regulation.

The maximum Pb concentration identified in this study reached 45,216 ppm, roughly 75 times higher than the current permissible limit of 600 ppm. This sample corresponded to one red paint applied on a metallic structure approximately 13 years ago, during the establishment of the Bici-Q public bicycle system. The persistence of such elevated Pb levels likely results from the prolonged use of the pigment “red lead” (Pb₃O₄), commonly incorporated in anticorrosive paints. Despite its protective efficiency, this compound poses severe toxic and carcinogenic risks^33,34^. In studies performed in the UK and South Africa in Pb-based paint of metallic playground equipment in public parks, Pb was found in concentrations ranging from 10 to 152,000 ppm (median = 451 ppm) and up to 10.4 mg cm^− 2^ (equivalent to > 50,000 ppm on a weight basis), respectively^35,36^. These results highlight the necessity of transitioning toward non-toxic alternatives capable of offering comparable anticorrosive performance. Currently, innovative lead-free pigment formulations are available that ensure effective corrosion resistance without the associated toxicity of Pb-based compounds^37^. Today, anticorrosive pigments with reduced toxicity are increasingly used in paint formulations.

Our results showed that yellow, orange and green pigments content the highest Pb values (39,613 ppm, 14,516 ppm, and 23,216 ppm, respectively), except for one red sample (45,216 ppm), suggesting that the main inorganic pigment used is lead-chromate^38^, which is imported by Ecuador from different countries, such as China, Colombia and Spain^39^. The most important Pb-based pigments in road paints for example, are with lead chromate (crocoite), PbCrO_4_, the mixed phase pigment of lead chromate and lead sulphate (lead sulphochromate; Pb(Cr, S)O_4_), and the mixed phase pigment of lead chromate, lead sulphate and lead molybdate (lead chromate molybdate sulphate; Pb(Cr,S,Mo)O_4_)^40^. Additionally, from 2022 to 2024, 114,066 kg of lead-plumbates (Pb₂PbO₄ and Pb₃O₄) were also imported in Ecuador^39^. In an investigation performed in traffic paint samples collected from roads in Ohio, USA, most yellow traffic paints had lead chromate, being a potential source for Pb contamination in surface water and groundwater^41^. While Turner 2023 showed similar results, in which yellow paints from road paints from eleven European countries, had Pb concentrations above 1,000 ppm^40^. The sustained demand for these compounds reveals a structural contradiction between regulatory intentions and industrial practice. Although the Ecuadorian legislation established a limit of 600 ppm for Pb in paints in 2012, enforcement remains weak, and monitoring mechanisms are limited. The continued importation of Pb oxides implies that some companies producing anticorrosive coatings and road-marking formulations have not yet transitioned to non-toxic or lead-free alternatives.

In the study conducted by Clark et al. (2009)^42^, paints obtained from retail stores in Ecuador exhibited an average Pb concentration of 31,960 ppm, reflecting extensive reliance on Pb-based pigments. Notably, in 2009 Ecuador lacked any legal restrictions on Pb content in paints. However, even in a later study performed by Romero-Estévez et al. (2019)^43^ encompassing 250 samples collected between 2015 and 2019, three to seven years after the regulatory limits were established, high Pb concentrations persisted in architectural, road-marking, and automotive coatings, revealing limited compliance with the established standards. Levels reached up to 21,044 ppm in synthetic enamels, while water-based paints also exhibited elevated Pb content (up to 12,715 ppm), with primarily intended use for interior household^43^. Despite the establishment of a Pb threshold limit in 2012, eradicating lead-based paints in Ecuador continues to pose a significant challenge. Both the production and trade of these toxic paints remain prevalent across the national market, reflecting weak regulatory enforcement and limited industrial transition toward safer alternatives.

Globally, similar situations have been documented. For instance, in Nepal, 93% of the tested paint samples exceeded 600 ppm, indicating that Pb-containing paints are still widely available for residential and other consumer uses in that country, often without any form of consumer warning^44^. These findings reflect a broader trend observed across developing countries where enforcement remains limited despite existing bans. Clark et al. (2014)^45^ revealed significant inconsistencies in labeling and manufacturing practices in India: one paint marketed as “no added lead” was found to contain 134,000 ppm of Pb, the highest concentration recorded in the study. Conversely, the fifth largest national brand consistently produced paints with Pb contents below 90 ppm, and in several cases below 15 ppm, confirming the technical and economic feasibility of producing lead-safe paints when proper formulations and quality controls are applied^45^, indicating that technological and economic barriers are not the primary obstacles to lead-free paint production.

Studies examining paints designed for architectural, artistic, and specialty applications across low and middle-income countries reveal substantial Pb contamination. Sargsyan et al. (2024)^46^ reported that, within Latin America, the proportion of samples exceeding Pb reference limits was greatest in Mexico (93%), followed by Colombia (31%), Peru (10%), and Bolivia (0%). The maximum Pb contents recorded in these samples ranged from 79,000 to 807,309 ppm, underscoring the persistence of Pb-based pigments in regional markets. It was also reported that other countries including Azerbaijan, Georgia, Nigeria, Tunisia, Turkey, Indonesia, Vietnam, and Indian States as Tamil Nadu, had 50% of paint samples with Pb concentrations above 90 ppm^46^.

At the outset of this study, it was hypothesized that yellow traffic paints, given their minimal direct human contact, would exhibit the highest Pb concentrations. Contrary to this expectation, some road paint samples had non-detectable Pb levels, whereas others showed markedly elevated concentrations, reaching up to 39,613 ppm. Also, repainting layers in some spots in road marking with high Pb levels (817 ppm) are evident, as shown in Fig. 8. These results clearly demonstrate that national normative are not fulfilled, even considering the highest threshold limit of Pb, with a weak enforcement by the Ecuadorian authorities. These Pb concentrations constitute non-compliance with of established regulatory limits, also the Pb levels found are incompatible with human health protection policies, and reflect a hazardous disregard for manufacturer accountability, particularly in terms of social and environmental responsibility.

One of the main limitations of this study concerns the absence of reliable data on the time elapsed since paint application. To address this, we focused on analyzing surfaces with the visual characteristics of recently applied coatings, classifying them as “new paints” (< 1 year). High Pb concentrations were detected in both old and new paints, implying that, as of 2025, certain manufacturers continue to incorporate Pb-based pigments into their formulations. The comparative evaluation of pXRF and FAAS results confirmed elevated Pb levels in paints purchased during 2024 (Table 1), suggesting ongoing production of Pb-containing paints in Ecuador. Nonetheless, several samples with undetectable Pb levels, found in both new and aged paints, demonstrate that some national industries have adopted responsible, lead-free manufacturing practices. In the last years, the access of substitutes for Pb additives in all types of paints and coatings has increased, mainly by European industry. AkzoNobel, a global paint manufacturer, eliminated the use of Pb in all their products in 2011, demonstrating that access to non-lead alternatives during manufacturing processes are possible, with performances equally or better over time than lead-paints^47,48^. These results emphasize the coexistence of compliant and non-compliant manufacturing practices, underscoring the need for systematic verification of paint formulations and regulatory enforcement.

International efforts, such as the Global Alliance to Eliminate Lead Paint, have set ambitious targets, yet progress remains slow. Effective interventions must include increased regulatory oversight, improved industry transparency, and public health campaigns aimed at raising awareness of lead exposure risks^18^. As reported by Akindele & Joseph (2024)^49^“cleaning up the environment after leaded paints have been removed from buildings is more expensive compared to the efforts required to eliminate or regulate lead compounds in paints by any government”.

Even though 72 painted sampling spots in the study area have high Pb concentrations ranging from 188 ppm to 39,613 ppm, not complying with the established thresholds, one of the most crucial problems is the weathering of paints as they break down from the painted surfaces, releasing Pb (II) ions into the soil^50^. Urban and peri‑urban soils act like long‑term sinks for potentially toxic pollutants including toxic metals, in which both natural and anthropogenic activities contribute to their distribution^51^. Several years ago, Takaoka et al. (2005)^52^ already showed that paint chips peeled off from playing equipment in playgrounds in Tokyo contributed to the increase soil Pb level with Pb concentrations between 15.2 and 237 mg/kg. Other studies have also reported that soils are likely to contain hazardous levels of bioavailable Pb from paint particles that have recently detached from the exterior surfaces^53^. In Ecuador, Armijos et al. (2021) measured Pb levels in residential windowsill dust, floor dust, and paint samples to identify environmental sources of Pb indoor exposure to school-age children and mothers. The mentioned study showed that home windowsill and floor dust samples strongly suggest that Pb-contaminated dust and soil may be important lead exposure sources, while peeling paint was not identified as a potential Pb source^54^. To the best of our knowledge, in Ecuador there are no studies related to the content of Pb in outdoor soils due to weathering of Pb-based paints.

Another important aspect, which is rarely discussed at the global level, concerns occupational exposure during the manufacturing, application, maintenance, repainting, and eventual removal or demolition of Pb-based paints and coatings. This dimension of exposure remains insufficiently addressed in both public health research and occupational safety regulations. Workers are susceptible for Pb poisoning^55^, leading into serious health effects that range from general symptoms as depression, generalized ache, and digestive signs, such as loss of appetite, stomachache, nausea, diarrhea, and constipation, to chronic conditions affecting cerebrovascular and cardiovascular systems, cognitive impairment, kidney disease, cancers, and infertility^56–58^. A small study conducted with 33 painters in Calceta (Manabí) found that 6.1% of the participants presented blood lead levels (BLLs) exceeding the safety limit^59^.

Regarding arsenic, our results showed that 52 painted spots had As levels between 106 ppm to 11,683 ppm. The highest values were found in one red sample, followed by yellow, green, and orange. Minerals containing As were widely used in artist pigments particularly during the 18th and 19th centuries^60^, providing pigments with different colors, e.g., green pigment CaCu(AsO_4_)(OH), the blue pigment obtained from processing cobaltite (CoAsS), red and yellow pigments (realgar, As_4_S_4_, and orpiment, As_2_S_3_, respectively)^61^. Arsenic has been confirmed as a carcinogenic agent in humans associated with skin and lung cancers, as well as affecting the activity of various enzymes involved in cellular respiration, glutathione metabolism, and DNA synthesis^62^. Despite its toxicity, As has been widely used for the production of antifouling coatings as a biocide agent for protection of the underwater parts of ships and submerged structures^63^. Scarce information is found related to As presence in new paints used on different surfaces. A study carried out in the EU in abandoned boats and ships maintenance areas reveal highly variable concentrations of toxic metals in paint fragments from boatyards, shipyards, slipways and boats abandoned on foreshores. Tin (Sn) and Pb were detected up to 40,000 ppm and 200,000 ppm, respectively, however, no As was measured^64^.

According to the Ecuadorian Technical Normative of traffic marking paints^65^, it should be free of arsenic. Unfortunately, in successive versions of this standard As is not considered to be controlled or restricted (INEN 1042-1, 2021). This finding alerts that other toxic elements are present in paints, and a wider vision should be encouraged to include As and other toxic metals into the regulatory standards and policies. Given our findings, it is imperative to expand paint sampling, not only in public areas but also for products available on retail shelves for direct purchase for diverse applications.

## Conclusions

According to our results, in the area under study in Quito, 102 painted spots contained Pb concentrations below the maximum threshold limit of 600 ppm, complying with the current regulation, which is in transition to new stricter values. On the other hand, 69 samples exceeded this limit up to 45,216 ppm, underscoring the weak government law enforcement to fulfill the low-lead requirements for paintings.

Even though the sampling area is small, significant information was obtained. It is evident that some Ecuadorian paint producers do not fulfill the established regulatory standards, therefore, national authorities must implement more stringent controls to restrict the import and utilization of lead-based pigments. Additionally, there is an urgent need to extend monitoring to indoor environments, particularly homes, nurseries, daycare centers, and playgrounds, where children may be exposed through peeling paint, settled floor dust, and windowsill dust.

Further analysis should be performed to assess Pb presence in soils due to paint weathering and chipping, as well as compliment more studies assessing the presence of As, considering that information of this metal in paints for different surfaces is scarce.

The present study establishes a valuable starting point for the development of evidence-based Pb mitigation and paint–management policies.

## Supplementary Information

Below is the link to the electronic supplementary material.

Supplementary Material 1

Supplementary Material 2

Supplementary Material 3

Supplementary Material 4

## Data Availability

Data will be made available on request.
