# Supplementary material for: The use of leaded paints in an urban neighborhood in Quito, Ecuador: A case study
Source: Sci Rep. 2026 Apr 25;16:19135. doi: 10.1038/s41598-026-48544-w (PMC13279780; doi:10.1038/s41598-026-48544-w)
Supplement: Supplementary file 1 — Supplementary Material 1 [file 41598_2026_48544_MOESM1_ESM.docx]

Supplementary Table S1. Lead and arsenic content in paint sample spots in the “university sector”, Quito, Ecuador.

| **Paint use** | **Color** | **Age of paint** | **Pb (ppm)** | **As (ppm)** |
| --- | --- | --- | --- | --- |
| Wall / art | Red | Spray paint (new) | 59 | 15 |
| Wall / art | White | Spray paint (new) | 15 | <LOD |
| Wall / art | Yellow | Spray paint (new) | 15 | <LOD |
| Wall / art | Red | Spray paint (new) | 71 | <LOD |
| Wall / art | Blue | Spray paint (new) | 48 | <LOD |
| Wall / art | Green | Spray paint (new) | 17 | <LOD |
| Wall / art | Green | Old | 10 | <LOD |
| Wall / art | Yellow | New | 9,810 | 1,152 |
| Wall / art | Red | New | 711 | <LOD |
| Wall / art | Green | New | 688 | <LOD |
| Wall / art | Yellow | Spray paint (new) | 6 | <LOD |
| Wall / art | Red | Spray paint (new) | 21 | 24 |
| Wall / art | Red | Spray paint (new) | 18 | <LOD |
| Wall / art | Green | Old | 11 | 7 |
| Wall / art | White | New | 23 | <LOD |
| Wall / art | Yellow | New | <LOD | <LOD |
| Wall / art | Yellow | New | 14 | 6 |
| Wall / art | Blue | New | 17 | <LOD |
| Wall / art | Green | Old | 9 | 5 |
| Wall / art | Blue | Old | 7 | <LOD |
| Wall / art | Yellow | Old | 15,073 | 3,972 |
| Road / art | Green | New | <LOD | 6 |
| Road / art | Green | Old | 9,062 | 2,096 |
| Road / art | Yellow | Old | 36,298 | 6,137 |
| Road / art | Red | Old | 47 | 13 |
| Road / art | Yellow | Old | 11,434 | 3,019 |
| Road / art | Green | Old | 8,079 | 2,230 |
| Road / art | Green | Old | 19 | <LOD |
| Road / art | Orange | Old | 17 | <LOD |
| Road / art | Blue | Old | 12 | <LOD |
| Road / art | Yellow | Old | 15 | <LOD |
| Road / art | Green | Old | 5,609 | 1,218 |
| Road / art | Red | Old | 566 | 107 |
| Road / art | Green | Old | 4,413 | 1,050 |
| Road / art | Yellow | Old | 13,001 | 2,727 |
| Road / art | Orange | Old | 14,516 | 2,419 |
| Road / art | Blue | Old | 167 | 37 |
| Road / art | Orange | Old | 161 | 38 |

| Road / art | Green | Old | 17,767 | 2,727 |
| --- | --- | --- | --- | --- |
| Metallic | Green | Old | 20,468 | 3,910 |
| Metallic | Yellow | New | 4,351 | 1,080 |
| Metallic | Green | New | 23,064 | 4,811 |
| Metallic | Orange | Old | 5,189 | 968 |
| Metallic | Orange | Old | 3,837 | 934 |
| Metallic | Black | Old | <LOD | <LOD |
| Metallic | Blue | New | <LOD | 48 |
| Metallic | Green | New | <LOD | 61 |
| Metallic | White | Old | 3,060 | 106 |
| Metallic | Green | Old | 2,587 | 626 |
| Metallic | Green | New | 6,142 | 1,642 |
| Metallic | Orange | New | 7,030 | 2,008 |
| Metallic | Red | Old | <LOD | <LOD |
| Metallic | Orange | New | 3,716 | 448 |
| Metallic | Red | Old | 45,216 | 11,683 |
| Metallic | Yellow | New | 39,414 | 11,527 |
| Metallic | Yellow | Old | 135 | 83 |
| Metallic | Green | Old | 4,430 | 1,221 |
| Metallic | Green | New | 1,041 | 65 |
| Metallic | Green | New | 4,302 | 1,220 |
| Metallic | Red | Old | 8,214 | 1,952 |
| Wall painting | Yellow | Old | 188 | 60 |
| Wall painting | Yellow | Old | 8 | <LOD |
| Wall painting | Red | New | 8 | 6 |
| Wall painting | Green | New | 10 | <LOD |
| Wall painting | White | New | <LOD | 14 |
| Wall painting | Green | Old | 285 | 52 |
| Wall painting | Yellow | New | 1,670 | 383 |
| Wall painting | Yellow | New | 138 | 15 |
| Wall painting | White | Old | 10 | <LOD |
| Wall painting | White | New | 258 | <LOD |
| Wall painting | Yellow | Old | 8 | 6 |
| Wall painting | Orange | New | 24 | 8 |
| Wall painting | Orange | New | 10 | 8 |
| Wall painting | Green | Old | 11 | 12 |
| Wall painting | White | Old | <LOD | 8 |
| Wall painting | White | Old | 33 | <LOD |
| Wall painting | Green | Old | 181 | <LOD |
| Wall painting | Yellow | New | 1,035 | <LOD |
| Wall painting | Black | New | 6 | 8 |
| Wall painting | White | New | 7 | <LOD |

| Wall painting | White | New | 7 | <LOD |
| --- | --- | --- | --- | --- |
| Wall painting | White | New | 8 | 11 |
| Wall painting | Blue | Old | 19 | <LOD |
| Wall painting | White | New | 75 | <LOD |
| Wall painting | Yellow | Old | 41 | 9 |
| Wall painting | White | Old | 9 | <LOD |
| Wall painting | Yellow | Old | <LOD | 8 |
| Wall painting | Yellow | Old | <LOD | 7 |
| Wall painting | Red | New | 8 | <LOD |
| Wall painting | Red | Old | 1,829 | <LOD |
| Wall painting | Red | Old | 10 | 10 |
| Wall painting | Green | New | 11 | 6 |
| Wall painting | Yellow | New | 18 | <LOD |
| Wall painting | Red | New | 23 | <LOD |
| Wall painting | Orange | Old | 769 | <LOD |
| Wall painting | Orange | New | 21 | <LOD |
| Wall painting | Red | New | 12 | 8 |
| Wall painting | Blue | Old | 414 | <LOD |
| Wall painting | Red | Spray paint (new) | 26 | <LOD |
| Wall painting | Orange | New | <LOD | 8 |
| Wall painting | Yellow | New | 156 | 17 |
| Wall painting | Yellow | Old | 23 | 6 |
| Wall painting | Green | Old | <LOD | <LOD |
| Wall painting | Red | Old | 10 | 7 |
| Wall painting | White | Old | <LOD | <LOD |
| Wall painting | White | Old | 7 | 6 |
| Wall painting | Yellow | Old | 6,701 | 2,055 |
| Wall painting | Orange | Old | 4,413 | 1,372 |
| Wall painting | Orange | New | 878 | 38 |
| Wall painting | Yellow | New | 9 | <LOD |
| Wall painting | Yellow | New | 8,645 | 256 |
| Wall painting | Orange | Old | 9 | 0 |
| Wall painting | Red | New | 27 | 13 |
| Wall painting | Orange | New | 6,062 | <LOD |
| Road marking | Yellow | Old | 21 | 7 |
| Road marking | Yellow | Old | 961 | <LOD |
| Road marking | Green | New | 1,073 | <LOD |
| Road marking | Yellow | New | <LOD | <LOD |
| Road marking | Yellow | Old | 1,032 | 213 |
| Road marking | Yellow | Old | 2,340 | 180 |
| Road marking | Yellow | Old | 26,090 | 1,501 |
| Road marking | Yellow | New | 6 | <LOD |

| Road marking | Yellow | New | 14 | <LOD |
| --- | --- | --- | --- | --- |
| Road marking | Yellow | New | <LOD | <LOD |
| Road marking | Yellow | New | 4,468 | 927 |
| Road marking | Green | New | 11 | <LOD |
| Road marking | Yellow | New | 817 | <LOD |
| Road marking | Yellow | Old | 3,387 | <LOD |
| Road marking | White | Old | 7 | <LOD |
| Road marking | Blue | Old | 5 | <LOD |
| Road marking | Green | New | 783 | 66 |
| Road marking | Yellow | New | <LOD | <LOD |
| Road marking | Yellow | Old | 12,040 | 2,345 |
| Road marking | Yellow | Old | 17,877 | 2,619 |
| Road marking | Yellow | New | <LOD | <LOD |
| Road marking | Yellow | Old | 5 | <LOD |
| Road marking | Yellow | Old | 18,601 | 3,608 |
| Road marking | Yellow | New | <LOD | <LOD |
| Road marking | Yellow | New | <LOD | <LOD |
| Road marking | Yellow | Old | 7,221 | <LOD |
| Road marking | Yellow | Old | 8,786 | <LOD |
| Road marking | Yellow | New | 7 | <LOD |
| Road marking | Yellow | Old | 10,462 | <LOD |
| Road marking | Yellow | Old | 10,336 | <LOD |
| Road marking | Yellow | Old | 13,426 | 1,889 |
| Road marking | Yellow | Old | 22,489 | 3,042 |
| Road marking | Yellow | Old | 24 | 6 |
| Road marking | Yellow | Old | 611 | <LOD |
| Road marking | Yellow | New | 13,775 | 2,623 |
| Road marking | Yellow | Old | 15,767 | 2,557 |
| Road marking | Yellow | Old | 24 | <LOD |
| Road marking | Yellow | Old | 19,261 | 3,395 |
| Road marking | Yellow | Old | <LOD | <LOD |
| Road marking | Yellow | New | 39,613 | 11,368 |
| Road marking | Yellow | New | <LOD | <LOD |
| Road marking | Green | New | 8 | <LOD |
| Road marking | Yellow | New | <LOD | 5 |
| Road marking | Yellow | New | 14,331 | 2,635 |
| Road marking | Yellow | Old | 10 | <LOD |
| Road marking | Yellow | Old | 21,262 | 3,486 |
| Road marking | Yellow | Old | 384 | 19 |
| Road marking | Yellow | New | 25 | <LOD |
| Road marking | Yellow | New | 17,161 | 2,164 |
| Road marking | Yellow | New | 25,010 | 1,776 |

| Road marking | Yellow | New | 6 | <LOD |
| --- | --- | --- | --- | --- |
| Road marking | Yellow | New | 8 | <LOD |
| Road marking | Yellow | New | 19,762 | 2,908 |
| Road marking | Yellow | New | <LOD | <LOD |
| Road marking | Yellow | New | 21,433 | 4,007 |
| Road marking | Yellow | New | 5,686 | 1,487 |
| Road marking | Yellow | New | 26,935 | 4,459 |
