# Supplementary material for: The use of leaded paints in an urban neighborhood in Quito, Ecuador: A case study
Source: Sci Rep. 2026 Apr 25;16:19135. doi: 10.1038/s41598-026-48544-w (PMC13279780; doi:10.1038/s41598-026-48544-w)
Supplement: Supplementary file 2 — Supplementary Material 2 [file 41598_2026_48544_MOESM2_ESM.docx]

Supplementary Table S2. Minimum, median and maximum lead levels in analyzed painted spots grouped according to colors.

| **Color** | **Number of analyzed painted spots** | **Lead content** | | |  |
| --- | --- | --- | --- | --- | --- |
|  |  | **Minimum (ppm)** | **Median (ppm)** | **Maximum (ppm)** |  |
| Orange | 16 | <LOD | 824 | 14,516 |  |
| Yellow | 77 | <LOD | 817 | 39,613 |  |
| Green | | 31 | <LOD | 285 | 23,064 |
| Red | | 19 | <LOD | 26 | 45,216 |
| Blue | 9 | <LOD | 17 | 414 |  |
| White | 17 | <LOD | 8 | 3,060 |  |
| Black | 2 | <LOD | <LOD | 6 |  |
