# Supplementary material for: The use of leaded paints in an urban neighborhood in Quito, Ecuador: A case study
Source: Sci Rep. 2026 Apr 25;16:19135. doi: 10.1038/s41598-026-48544-w (PMC13279780; doi:10.1038/s41598-026-48544-w)
Supplement: Supplementary file 3 — Supplementary Material 3 [file 41598_2026_48544_MOESM3_ESM.docx]

Supplementary Table S3. Minimum, median and maximum lead levels in analyzed painted spots grouped according to use.

| **Surface/use** | **Number of analyzed painted spots# of** | **Lead content** | |  |
| --- | --- | --- | --- | --- |
|  |  | **Minimum (ppm)** | **Median (ppm)** | **Maximum (ppm)** |
| Metallic | 21 | <LOD | 4,302 | 45,216 |
| Road / art | 18 | <LOD | 2,490 | 36,298 |
| Road marking | 57 | <LOD | 817 | 39,613 |
| Wall/art | 21 | <LOD | 17 | 15,073 |
| Wall painting | 54 | <LOD | 15 | 8,645 |
