# Supplementary material for: The use of leaded paints in an urban neighborhood in Quito, Ecuador: A case study
Source: Sci Rep. 2026 Apr 25;16:19135. doi: 10.1038/s41598-026-48544-w (PMC13279780; doi:10.1038/s41598-026-48544-w)
Supplement: Supplementary file 4 — Supplementary Material 4 [file 41598_2026_48544_MOESM4_ESM.docx]

Supplementary Table S4. Minimum, median and maximum lead levels in analyzed painted spots grouped according to age of paint.

| **Age of paint** | **Number of analyzed painted spots# of** | **Lead content** |  |  |
| --- | --- | --- | --- | --- |
|  |  | **Minimum (ppm)** | **Median (ppm)** | **Maximum (ppm)** |
| > 1 year | 86 | <LOD | 384 | 45,216 |
| New paint | 75 | <LOD | 23 | 39,613 |
| Spray (new) | 10 | 6 | 18 | 71 |
